# Technological Devices Improving System of Translating Languages: What About their Usefulness on the Applicability in Medicine and Health Sciences?

**DOI:** 10.5935/1678-9741.20150087

**Published:** 2015

**Authors:** Adilia Maria Pires Sciarra, Fernando Batigália, Marcos Aurélio Barboza de Oliveira

**Affiliations:** 1 Faculdade de Medicina de São José do Rio Preto (FAMERP), São José do Rio Preto, SP, Brazil; 2 UNIFEV, Votuporanga, SP, Brazil

**Keywords:** Medicine, Health Sciences, Technology, Online Systems, Translations.

## Abstract

**INTRODUCTION:**

In a world in which global communication is becoming ever more important and in which English is increasingly positioned as the pre-eminent international language, that is, English as a Lingua Franca refers to the use of English as a medium of communication between peoples of different languages. It is important to highlight the positive advances in communication in health, provided by technology.

**OBJECTIVE:**

To present an overview on some technological devices of translating languages provided by the Web as well as to point out some advantages and disadvantages specially using Google Translate in Medicine and Health Sciences.

**METHODS:**

A bibliographical survey was performed to provide an overview on the usefulness of online translators for applicability using written and spoken languages.

**RESULTS:**

As we have to consider this question to be further surely answered, this study could present some advantages and disadvantages in using translating online devices.

**CONCLUSION:**

Considering Medicine and Health Sciences as expressive into the human scientific knowledge to be spread worldwidely; technological devices available on communication should be used to overcome some language barriers either written or spoken, but with some caution depending on the context of their applicability.

**Table:** 

**Abbreviations, acronyms & symbols**	
GT	= Google Translate
PI	= Professional interpreters
MT	= Machine translation
RSS	= Really Simple Syndication / Rich Site Summary
WWW	= World Wide Web

## INTRODUCTION

Advances in technology are one of the main reasons that globalization has escalated in the past decades. In information and communication technology, innovations have become smaller in size, more efficient and often more affordable. The Internet and the development of digital technology (computerbased technology) in particular have made the most significant impact in the field of information and communication technology. The Internet is essentially a network of computers across the world which is linked through global telecommunications. Although it was originally only used by defense personnel in the United States, easy access to computers and related technology have made using the Internet a common activity in more recent times^[1]^.

The World Wide Web (www) is a collection of interconnected documents, which are accessible using the Internet. It enables people from almost anywhere in the world to access information on almost any topic from shopping to weather forecasts; and from research to downloading music and movies. In addition to the Internet, global media networks (corporations which include television and media companies with branches in multiple countries) also bring news and information about current events to people all over the globe. It is now possible for someone in Australia to pick up a copy of an American fashion magazine, or for someone in the United States or in Brazil. Therefore, the growing influence of the Internet has been reflected in the appearance of software products specifically for translating Web pages aiming to break language barriers^[2]^.

### Machine translation: a brief history

Computer programs (software) for translation of natural languages, commonly and traditionally called "machine translation" (MT) or, in non-English speaking countries, "automatic translation" (traduction automatique, automaticheskij perevod) were initially developed for translations of scientific and technical documents (its beginnings in the 1940s). Since the mid-1990s, the Internet has exerted a powerful influence on MT development. First, there has been the appearance of MT software products for translating specifically Web pages and electronic messages offline led by Japanese companies. Afterwards, many Internet-based online translation services were provided for this on-demand translation such as Alta Vista site, Reverso systems, LogoMedia and Pars. Some services offered post-editing human translators (revisers), at extra cost accessed through "MT portals", that is, independent services offering a range of translation systems from one or more system companies; most of them free of charge. Basically in Science and Technology, the demand for translation has almost exceeded the capacity of the translator professional, as a consequence, for this growing need, the Internet has had a growing influence reflected in the appearance MT software products for translating languages^[3]^.

It is now clear that different types of MT systems are required to meet widely differing translation needs. Those identified so far include the traditional MT systems for large organizations, usually within a restricted domain; the translation tools and workstations designed for professional translators. The advent of the cheap personal computer systems for occasional translations; the use of systems to obtain messages for the purposes of surveillance or information gathering; the use of MT for translating electronic messages (electronic mail and Web pages, in particular); systems for monolinguals to translate standard messages into unknown languages; systems for speech translation in restricted domains were included into these systems^[4]^.

It is equally clear that as MT systems of many varieties become more widely known and have been using the range of possible translation needs and possible types of MT systems. These will also become wider and stimulate further research and development, quite probably in directions not yet envisioned^[4]^.

To sum up, the related development of computer-based translation tools was primarily the use of dictionaries and glossaries to translate technical texts, and later, the translation databases and translator workstation such as the Google Translate (GT), Bing Translate, Yahoo, Babel Fish e Systran were developed influenced by the Internet.

### Google Translate

Like most online translation tools, GT works on something called Statistical Machine Translation which basically gathers loads of data over time to statistically offer the best combination of translation in any given language pair.

GT is one of the most used machine translation tools online. It is a free virtual service at no-cost. GT offers its services subsidiary of Google Inc. Alphabet instant translation of texts, websites, speech, images, or real-time video from one language into another; translations are instantaneous and for immediate use. It offers a web interface, mobile interfaces for Android and iOS, and an API that developers can use to build browser extensions, applications and other software. Since September 2015, GT supports 90 languages at various levels and serves over 200 million people daily. The company introduced its own translation software in 2007 (7^th^ October), as before used by SYSTRAN translator, used by other translation services such as Babel Fish (Altavista), the translator of AOL, Yahoo and MSN^[5]^.

Recently, GT has increasingly implemented improvements to its mechanism, thus making it one of the leading services in idiomatic translation. The team at Google is working to expand available languages and capabilities in computing power for translating conversations. The company began rolling out a new version of a free GT application that, in part lets people point Android or Apple smartphones at signs, menus, recipes or other material written in French, German, Italian, Portuguese, Russian, or Spanish and see them in English.

One can instantly translate text using a camera, so it is an easier way to navigate street signs in the Italian countryside, for example, or decide what to order off a Barcelona menu. This feature was built on Word Lens technology that Google acquired last year when it bought Quest Visual, that is, Word Lens which use video mode in smartphone cameras to scan scenes, identify writing and then display it as if it were written in English^[6]^.

We aimed to present an overview on devices for translating languages provided mainly by the Web which have been strengthening communication among peoples in a virtual globalized world. Moreover to point out their usefulness on the applicability particularly on Medicine and Health Sciences regarding both skills of communication: spoken and written.

## METHODS

This research was carried out by means of a bibliographical survey to access the development of technological translating devices offered by the WEB service since their creation up to nowadays. An issue was addressed to readers for reflecting about the usefulness on the applicability of these tools in Medicine and Health Sciences. Online Translators and GT were the main important terms surveyed to reach related contents for the goal of this report.

## DISCUSSION

At first, taking into account communication as the cornerstone of Medicine even between doctors and their patients or the growing of information and knowledge into the field of Health Sciences mainly written in English; translators online such as GT can be considered useful tool when language is a barrier in communication; that is, GT presents a possible solution to this problem. As an extension of their widespread integration of speech-to-text throughout the android platform, Google created a language app centered around a straightforward concept; enter one language, output in another.

This device still remains the most easily available and free initial mode of communication in general. The increased availability and use of the Internet have facilitated online communication between physicians and patients when the patient is not physically present at the physician's place of practice. Communication by using translators may be directly related to the provision of patient care or may be used for transmitting more general information for administrative, educational or health promotional purposes. These were primarily concerned with the two most common vehicles of online communications: email and physician websites^[7]^.

In the evaluation of the accuracy and usefulness of GT in translating ten commonly English medical statements used in communications between patients and doctors; Davies^[8]^ has claimed limited usefulness for these medical phrases (57.7% accuracy). The author's recommendation is that it should not be trusted for important medical communication based on this assessment.

In another study, GT was tested as an alternative for professional interpreters' (PI) work since this was not available at that time. The translation of 20 standardized sentences from a neonatal doctor-/nurse-relative interview from German to English, Portuguese and Arabic, using GT was not effective to overcome language barriers in neonatal medicine. The assessment on translation by GT showed an average of 42% of the sentences was correctly translated concerning grammar and content. The proportion of incorrectly translated sentences varied between 45-70%. By simplification, another 23% were translated correctly.

Translations by GT were often incorrect in content and grammar. The supposition was that the design of GT, which is a statistical translation engine, might be an explanation for this phenomenon. Presently, GT cannot guarantee unambiguous translations and cannot substitute PIs; only in particular circumstances, the use of GT or similar engines may be justified. The recommendation proposed was for future use of electronic translation services to compile a catalogue of these standardized sentences containing central information, and afterwards they should be translated into defined foreign languages without misinterpretation or loss of information^[9]^.

On top of updating reports, GT team is continuously working to improve the quality of the translations themselves and to add new languages. A year ago GT Community was launched, that is, a place for multilingual people from anywhere in the world to provide and correct translations. The millions of language lovers who have already pitched in more than 100 million words so far have been updating its translations for over 90 language pairs, and plan to update many more as the community grows^[10]^.

Computing power for translating conversations comes from Google servers, so connections to the Internet through WiFi or telecom carriers are needed. The team at Google is working to expand available languages and capabilities, thus they still have lots of work to do: more than half of the content on the Internet is in English, but only around 20% of the world's population speaks English. Today's updates knock down a few more language barriers, helping people to communicate better and get the information they need.

This latest innovation of GT could also help immigrants and refugees with limited language proficiency when they are settled in a new society, with new language and new culture. The consequences of language barriers affect the ability of these new residents to live successfully in the country where language is a pathway to their integration^[11]^.

To reach that level of accuracy, intelligent machines have to not just convert each word into a new language, but analyze entire phrases for context and infer their meaning before offering up a translation the way a human interpreter does. If a product can offer accurate real-time language translation with an augmented device that someone would actually want to use, that'll be a seriously world-changing thing. Forget love, technology is shaping up to be the universal language.

## CONCLUSION AND FINAL CONSIDERATIONS

GT shows a lot of promise, and hopefully it can be useful either for the tourists or the physicians who want to eliminate some gaps of language misunderstanding as in spoken or written communication. In the clinical setting where spoken language is used between physician and patient, GT has an extension of widespread integration of speech-to-text throughout the android platform: Android OS. However, both the physician and the patient would be better served by a medical dictionary or a professional translator, if available. Google has big plans for real-time translation, and they expect it to vastly improve in the next few years. As it stands, however, the medical professional cannot afford to compromise the patient care by early-adoption of this technology.

Online translators have already taken a big step in their codes, but are still far from what would be ideal. The main reasons are: not everything is translated with the service, thus, a comprehensive translation of the text will not be provided. Not every language is offered. It does not give natural, fluently readable translations; GT gives a mechanical and direct translation of words. Context is not part of the equation: GT will not know the context of the content. For example, who is the audience, what are their ages, education level and cultural sensitivities? In addition, what is the content being used for? Eg. for a speech, marketing copy, website, white paper, instructional content or something else?

It increasingly becomes a tool for professional translators who improve the text and take questions through the services. In addition, machine translation is recommended for quick translations, on websites and, in general texts. Considering the rigidity of scientific written language, one should take into mind the words into their context in such model of communication. The recommendation is to be aware of the multiple meaning a word can present (POLYSEMY). In his book "Linguagem Médica", Dr Joffre M de Rezende (http://www.jmrezende.com.br/) pointed out the traps the translator could fall if he does not consider this characteristic of the language. To consult a standard-bilingual specialized dictionary for searching entries and their definition-translations could be the first solution for any doubt on mistranslation considering hazard statements in Medicine.

Take a look at OneLook Dictionary Search (http://www.onelook.com), an online dictionary (monolingual or bilingual). If you need to type a word, phrase or acronym and the site does a search in more than a thousand dictionaries. It is called the Google of dictionaries. Among them, the best known are as Macmillan Dictionary, Merriam-Webster's Online Dictionary, Collins Dictionary, Cambridge Advanced Learner's Dictionary, American Heritage Dictionary of the English Language, Compact Oxford English Dictionary, etc. Results are grouped by category. For example, if you type stem cell, you see a list of generic dictionary and then the medicals. If you enter interest rate, again we have the generic followed by the economy. Then you choose which construction or consulting. It follows some online addresses for Consulting.

TheFreeDictionary.com (http://pt.thefreedictionary.com/) allows you to create your own personal homepage by adding and removing, dragging and dropping, and "using or losing" existing content windows. In addition, you can add your own bookmarks, weather information, horoscope, and RSS feeds (Really Simple Syndication or Rich Site Summary) from anywhere on the web.

The Visuwords online graphical dictionary and thesaurus (http://www.visuwords.com/) is indicated to look up words to find their meanings and associations with other words and concepts. To produce diagrams reminiscent of a neural net. To earn how words associate. To enter words into the search box to look them up or double-click a node to expand the tree. Click and drag the background to pan around and use the mouse wheel to zoom. Hover over nodes to see the definition and click and drag individual nodes to move them around to help clarify connections.

Visuwords uses Princeton University's WordNet, an open source database built by University students and language researchers combined with a visualization tool and user interface built from a combination of modern web technologies.

All these online devices are available as a free resource to all people's demand in the Web.

The second solution is also given by the democratic Web: the Linguee (http://www.linguee.com/) is a unique translation tool combining an editorial dictionary and a search engine with which one can search billions of bilingual texts for words and expressions. Compared to traditional online dictionaries, Linguee contains about 1,000 times more translated texts, which are displayed in full sentences. Linguee shows translations for expressions such as "strong evidence, strong relationship or strong opinion", and even for rare expressions or specific technical terms.

The Linguee search results are divided into several sections. In the top section you are shown results from the reliable editorial dictionary. Professional editors have been working continuously on adding new entries and enhancing the quality of the dictionary. This provides a quick overview of various translations of your search term. Below you are shown example sentences from other sources to give you an idea of how your search term has been translated in context.

But the question still remains: to give trust? In terms. There is still no complete good to replace human translation; based on knowledge, and a good deal of effort and a lot of interest in keeping an eye on the evolution of these online devices are recommended.

**Table:** 

**Authors' roles & responsibilities**	
AMPS	Conception and design; manuscript writing or critical review of its contents; final approval of the manuscript
FB	Manuscript writing or critical review of its contents; final approval of the manuscript
MABO	Manuscript writing and critical review of its contents; final approval of the manuscript
